# Structural Basis of Cytotoxicity Mediated by the Type III Secretion Toxin ExoU from *Pseudomonas aeruginosa*

**DOI:** 10.1371/journal.ppat.1002637

**Published:** 2012-04-05

**Authors:** Claire Gendrin, Carlos Contreras-Martel, Stéphanie Bouillot, Sylvie Elsen, David Lemaire, Dimitrios A. Skoufias, Philippe Huber, Ina Attree, Andréa Dessen

**Affiliations:** 1 Bacterial Pathogenesis Group, Institut de Biologie Structurale (IBS), Université Grenoble I, Grenoble, France; 2 Commissariat à l'Enérgie Atomique (CEA), Grenoble and Cadarache, France; 3 Centre National de la Recherche Scientifique (CNRS), Grenoble and Cadarache, France; 4 Bacterial Pathogenesis and Cellular Responses, iRTSV, Université Grenoble I, Grenoble, France; 5 INSERM UMR-S 1036, Biology of Cancer and Infection, Grenoble, France; 6 Laboratoire des Interactions Protéine Métal, IBEB, Université Aix-Marseille II, Saint Paul Lez Durance, France; 7 Viral Infection and Cancer Group, IBS, Grenoble, France; University of Michigan, United States of America

## Abstract

The type III secretion system (T3SS) is a complex macromolecular machinery employed by a number of Gram-negative pathogens to inject effectors directly into the cytoplasm of eukaryotic cells. ExoU from the opportunistic pathogen *Pseudomonas aeruginosa* is one of the most aggressive toxins injected by a T3SS, leading to rapid cell necrosis. Here we report the crystal structure of ExoU in complex with its chaperone, SpcU. ExoU folds into membrane-binding, bridging, and phospholipase domains. SpcU maintains the N-terminus of ExoU in an unfolded state, required for secretion. The phospholipase domain carries an embedded catalytic site whose position within ExoU does not permit direct interaction with the bilayer, which suggests that ExoU must undergo a conformational rearrangement in order to access lipids within the target membrane. The bridging domain connects catalytic domain and membrane-binding domains, the latter of which displays specificity to PI(4,5)P_2_. Both transfection experiments and infection of eukaryotic cells with ExoU-secreting bacteria show that ExoU ubiquitination results in its co-localization with endosomal markers. This could reflect an attempt of the infected cell to target ExoU for degradation in order to protect itself from its aggressive cytotoxic action.

## Author Summary

*Pseudomonas aeruginosa* is a leading cause of nosocomial infections and is a particular threat for cystic fibrosis and immunodepressed patients. One of the most aggressive toxins in its arsenal is ExoU, injected directly into target cells by a needle-like complex located on the surface of the bacterium, the type III secretion system. *P. aeruginosa* strains that express ExoU cause rapid cell death as a consequence of the membrane-destruction (phospholipase) potential of the toxin. In this work, we report the three-dimensional structure of ExoU in complex with a partner molecule, SpcU. ExoU contains three distinct regions, and the fold suggests how ExoU binds to the membrane or other molecules within the target cell and becomes activated. In addition, we also show that once it is translocated into the cell, ExoU co-localizes with intracellular organelles of the endosomal pathway, potentially in an attempt of the target cell to destroy the toxin. This work provides new insight into the cellular destruction mechanism of this aggressive toxin and could be a basis for the development of new inhibitors of *P. aeruginosa* pathogenesis.

## Introduction

Type III secretion systems (T3SS) are needle-like, membrane-anchored, multi-component complexes that enable a number of pathogenic bacteria to inject effectors from the cytosol directly into the cytoplasm of eukaryotic cells [1]–[6]. T3SS are widespread among Gram-negative bacteria, and although the structure of the T3SS apparatus itself can display notable similarities amongst different bacterial species [5], [7], the nature of translocated effectors are widely different. Many T3SS-translocated proteins have been shown to modulate cellular functions, i.e. by mimicking protein kinases, phosphatases, GTPase activating proteins, or ubiquitin ligases, or by covalently modifying target proteins through phosphorylation or acetylation. The consequences to the target cell may range from modifications of the cytoskeleton to membrane disruption and apoptosis [8], underlining the key nature of T3SS for extreme pathogenesis in a number of systems.

*Pseudomonas aeruginosa* is a leading cause of nosocomial infections and is a major threat to cystic fibrosis patients and others with impaired immune defenses. It carries a T3SS whose upregulation during acute phases of infection is directly related to poor patient prognosis [9], [10], and which translocates four effectors, namely exoenzymes S, T, U, and Y [11]–[13]. ExoS and ExoT are bifunctional molecules with GTPase-activating (GAP) and ADP-ribosyltransferase activities essential for the inhibition of bacterial internalization and epithelial cell migration [14]–[19]. ExoY is an adenylate cyclase reported to play a role in actin cytoskeleton disruption and cause cell rounding [20]–[22]. However, it is ExoU which is the most detrimental toxin injected by the T3SS of *P. aeruginosa*. ExoU is expressed by approximately 30% of clinical strains, 90% of which cause acute illness [10], [11]. It is encoded on a pathogenicity island together with its cognate chaperone SpcU, which is required for ExoU's efficient secretion from the bacterial cytoplasm [23], [24]. ExoU is a 687-residue protein that, once translocated through the T3SS, induces cytotoxic effects leading to rapid necrotic cell death; *exoU* knockout *P. aeruginosa* strains display greatly decreased virulence in mouse models of acute infection [25], [26]. In clinical settings, ExoU-expressing *P. aeruginosa* strains lead to poor patient prognosis, since the toxin causes acute lung epithelial injury and is linked to the development of septic shock [10], [18], [27]–[30]. To date, the precise mechanism underlying ExoU's significant potency for cellular destruction has remained unclear.

Notably, ExoU has been shown to carry phospholipase activity with broad substrate specificity which relies on an essential catalytic dyad (Ser142/Asp344) [24], [31]–[33], as is the case for other phospholipases. Enzymes with PLA_2_ activity hydrolyze the *sn*-2 ester bond of phospholipids, and thus play a role in membrane disruption, fatty acid release, and in many cases, signal transduction [34]–[36]. Interestingly, expression of ExoU in yeast causes rapid fragmentation of the vacuolar compartment, suggesting that eukaryotic membranes are the major targets for the toxin [33]. It is of note that the C-terminus of ExoU is critical for its *in vitro* phospholipase activity and cellular cytotoxicity [32], [37]–[40]. In addition, pre-incubation with cellular extracts has been shown to be essential for detection of phospholipase activity *in vitro* [33], [41], indicating the requirement for host eukaryotic cofactors in order for ExoU to exert its phospholipase activity on lipidic substrates. Eukaryotic Cu/Zn superoxide dismutase (SOD1) and other ubiquitinated proteins, as well as ubiquitin itself, have been suggested as being potential activators of the toxin [42]–[44].

The extreme toxicity of ExoU has limited functional studies of the wild type form, necessitating the employment of a catalytically-inactive mutant in cellular assays [31]–[33]. Trafficking studies of the ExoU-Ser142Ala mutant in eukaryotic cells have revealed that, upon translocation through the T3SS, ExoU is initially targeted to the plasma membrane (PM). The membrane localization potential of ExoU resides in its C-terminal domain (specifically, in residues 679–683). ExoU trafficking to the PM enables its ubiquitination on Lys178 [39], but this modification has only a modest effect on ExoU turnover and an ExoU-K178R-expressing mutant strain displays cytotoxicity levels that are similar to those of the wild-type. These observations attest to the complex fate of ExoU in target cells, which implies the coordination between membrane targeting, phospholipase activity, activation by cofactors, and ubiquitination.

In order to understand the multi-faceted behavior of ExoU, we solved its crystal structure, which was achieved in the presence of its chaperone, SpcU. ExoU forms a 1∶1 complex with SpcU, and folds into three distinct domains, which fulfill catalytic, bridging, and membrane-binding functions. The active site of ExoU is localized within an α/β hydrolase fold in a cleft sheltered by flexible loop regions, thus suggesting an inactive conformation that would require a structural modification to allow access of the nucleophilic serine to the lipidic substrates on the plasma membrane. The localization of Lys178 in proximity to the active site region implies that such structural rearrangements could also facilitate ubiquitination of ExoU by PM-localized E3 ligases. Ubiquitinated ExoU co-localizes with markers of endosomal compartments both upon transfection of cultured cells and infection of eukaryotic cells by an ExoU-expressing *P. aeruginosa* strain. This reflects a potential attempt of the cell to eliminate the toxin by targeting it for lysosomal destruction.

## Results

### SpcU's type IA fold stabilizes the N-terminus of ExoU

**Figure 1:**
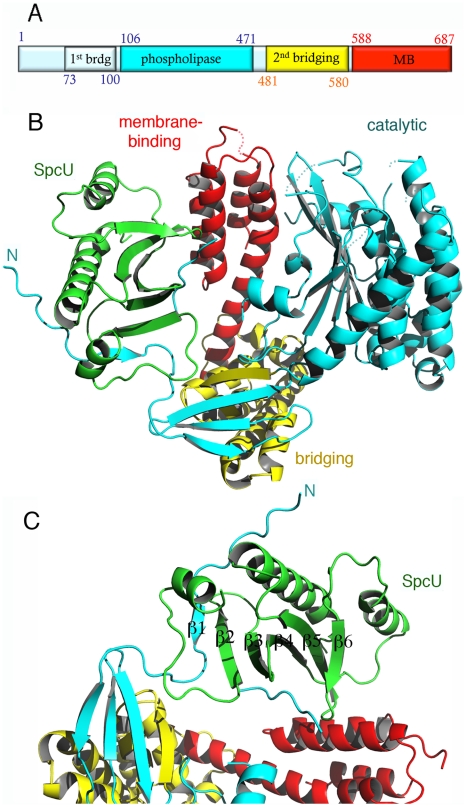
The crystal structure of ExoU in complex with its chaperone SpcU. (A) Schematic diagram of the ExoU construct used in this work and limitations of different domains. The first and last ExoU residues observed in the electron density map are Gly53 and Glu685. The catalytic domain is inserted within the first and second bridging subdomains. (B) ExoU folds into three distinct domains and its N-terminal β-strand is paired with SpcU's β-sheet. The bridging domain (yellow) contains elements that are both N-terminal and C-terminal to the catalytic domain (blue). The membrane-binding domain makes few contacts with the other domains of ExoU. (C) SpcU (green) is a type IA T3SS chaperone. The N-terminus of ExoU, packs as a β-strand against the central 5-stranded SpcU sheet. For clarity, only the asymmetric unit is shown.

The structure of the ExoU(30–687)∶SpcU(1–127) complex was solved to a resolution of 2.94 Å by performing a SAD experiment on the selenium edge at the ESRF synchrotron in Grenoble (Supplementary Table I), and harbors a 1∶1 complex in the asymmetric unit. This stoichiometry is unusual, since SpcU has a typical type IA T3SS chaperone fold (Figure 1), and complexes between T3SS effectors and type I chaperones typically associate with stoichiometries of 1∶2 [3], [5], [45]. In order to determine the precise stoichiometry of ExoU∶SpcU *in vitro*, we performed native mass spectrometry measurements of the purified complex, which allowed us to identify a mixture of two distinct stoichiometric species, 1∶1 and 1∶2. Interestingly, close analysis of the structure reveals how both stoichiometries are possible in the crystal lattice.

SpcU is a typical type IA chaperone, being composed of a curved, 5-stranded sheet flanked by α-helices. SpcU displays two hydrophobic patches that recognize the effector. One involves the extremity of the 5-stranded β-sheet, which binds a short β-strand located at the N-terminus of ExoU (residues 62–64), thus generating a 6-stranded sheet (Figures 1c and S1). This binding region corresponds to the β motif that is also present in SipA∶InvB, SptP∶SicP, YopH∶SycH, and YopE∶SycE complexes [46]–[48]. An additional hydrophobic region of SpcU, located at the center of the β-sheet, binds a region of ExoU that is partly disordered in the electron density map. It is conceivable that this region corresponds to the flexible N-terminus of ExoU that is stabilized in a symmetry-related SpcU molecule. This becomes more evident upon the generation of an ExoU∶SpcU dimer (Figure S1c) based on the possible biological assemblies calculated by PISA (http://www.ebi.ac.uk/msd-srv/prot_int/picite.html), as well as by symmetry within the C2 cell, which suggests that the flexible N-terminus of ExoU in fact wraps around the crystallographic SpcU dimer by employing two different hydrophobic patches on the chaperone (the “front" and the “back", Figures S1b, S1c). It is of interest that the effectors in the abovementioned 1∶2 complexes display N-termini that surround their respective chaperone dimers in comparable fashion. Thus, it is conceivable that *in vitro*, both ExoU∶SpcU stoichiometric forms exist in equilibrium, but the 1∶1 form, presented here, is the one that crystallizes in the asymmetric unit of a C2 cell. Notably, the complex between the *Yersinia pestis* chaperone SycH and a fragment from the effector YscM2 also crystallized with 1∶1 stoichiometry, with the effector wrapped around the chaperone in a similarly extended conformation [49].

This association presumably ensures that the N-terminus of ExoU is ready for translocation through the T3SS, and is also in agreement with data indicating that the minimal domain required for cytotoxicity begins in a region residing between residues 52 and 100 [50]; in fact, the last residue in the N-terminal region of ExoU that makes contacts with SpcU is Ser65.

### ExoU folds into three independent domains

Immediately following the SpcU-bound N-terminus of ExoU is the first part of the bridging domain, which precedes the catalytic region. The bridging domain of ExoU consists of two subdomains: an N-terminal 4-stranded region (cyan and yellow in Fig. 1b) and an all-helical section (yellow in Figures 1b and S2). Notably, the ExoU sequence that immediately follows the N-terminal, SpcU-associated region contributes 3 β-strands to the 4-stranded sheet of the first subdomain (β2–β4). The catalytic domain is intercalated at this point; β11 then complements the first subdomain, and this is followed by the all-helical C-terminal subdomain (residues 480–580). The bridging domain itself has no significant structural similarity with any known folds, as determined by the DALI server (http://ekhidna.biocenter.helsinki.fi/dali_server/). Due to its intercalated position between catalytic and membrane-binding domains, it is possible that its function is uniquely to serve as a ‘platform’ to place both functionally critical domains in close proximity.

**Figure 2:**
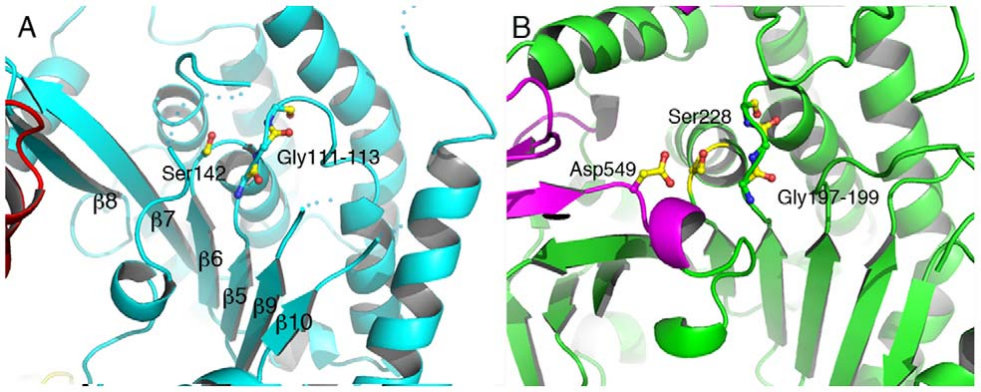
The catalytic domain of ExoU displays an α/β hydrolase fold. (A) Ser142 is in close proximity to the Gly111–113 elbow, which acts as an oxyanion hole for the phospholipase-catalyzed reaction. The second member of the Ser-Asp catalytic dyad, Asp344, is located in a flexible region that is not traceable in the electron density map. The overall arrangement of the core region of ExoU's catalytic domain is reminiscent of that of human cPLA_2_ (B), which also harbors the catalytic aspartate in a moveable ‘cap’ (in violet).

Residues 106–471 delimit the catalytic, phospholipase domain, which is composed of an α/β hydrolase fold with a 6-stranded central β-sheet surrounded by 10 α-helices (Figures 1 and 2a). The sheet displays a superhelical twist with β strands 8 and 10 lying at approximately 90° to each other. The catalytic serine (Ser142) is located at an elbow between β6 and the following helix, in a funnel-like region surrounded by a number of flexible loops. A triple glycine sequence (Gly111, 112, 113), in close proximity, provides its backbone atoms as an oxyanion hole that potentially stabilizes the charge of the transition state intermediate in the phospholipase reaction (Figure 2a). This structural arrangement is highly similar to that of a well-studied and potent phospholipase, human cPLA_2_, whose structure also carries a twisted β-sheet that prominently displays the catalytic serine in proximity to the oxyanion hole [51] (Figure 2b). Additionally, the structure of cPLA_2_ harbors an active site ‘cap’ – a presumably flexible region that must be displaced in order to enable substrate access into the active site. It is precisely in this ‘cap’ region that is located the catalytic aspartate residue, essential for phospholipase activity [51]. In the structure of ExoU, this region is also found to be flexible, and the catalytic aspartate (Asp344) is not traceable in the electron density map (Figure 2a), suggesting not only that ExoU could also harbor a ‘cap’ region, but that the structure presented here is in the ‘open ’, and potentially inactive, conformation.

**Figure 3:**
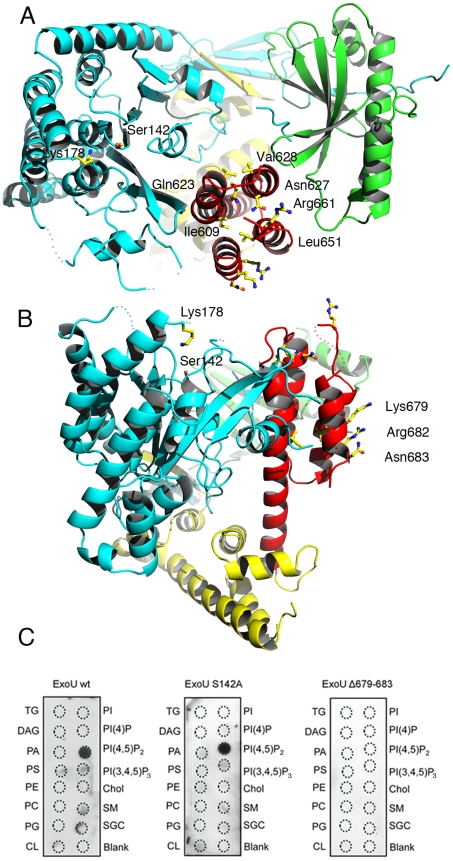
Membrane-binding, ubiquitination, and catalysis are structurally intertwined. (A) Mutations in the C-terminus of ExoU [31], [32], [37]–[40] that abrogate membrane binding and cytotoxicity are all located within the four-helical bundle (red). This region is located on the same face of the molecule as the catalytic region, as well as Lys178 (left). (B) Residues 679–683 are exposed to solvent, in a potential appropriate orientation for bilayer recognition. (C) Protein-lipid overlays of wt ExoU, ExoU-S142A and ExoU-Δ679–683 reveal that the C-terminal region is essential for a strong, specific interaction with PI(4,5)P_2_. TG, triglyceride; DAG, diacylglycerol; PA, phosphatidic acid; PS, phosphatidylserine; PE, phosphatidylethanolamine; PC, phosphatidylcholine; PG, phosphatidylglycerol; CL, cardiolipin; PI, phosphatidyl-inositol; PI(4)P, phosphatidylinositol 4 phosphate; PI(4,5)P_2_, phosphatidylinositol (4,5) di-phosphate; PI(3,4,5)P_3_, phosphatidylinositol (3,4,5) triphosphate; chol, cholesterol; SM, sphingomyelin; SGC, 3 sulfogalactosylceramide. Blank=no lipid spotted.

Interestingly, Lys178, shown to be the target for ubiquitination once ExoU upon membrane localization, is located within the phospholipase domain, in close proximity to the active site (Figures 3a, 3b). Its side chain is located approximately 7 Å away from the active site serine, in a region that displays high flexibility. This strategic localization could favor the ability of this particular lysine residue to become ubiquitinated (see below).

The C-terminus of ExoU has been shown to play a key role in membrane targeting, and numerous modifications of this region that have an impact ExoU's cytotoxicity have been described, including the deletion of residues 679–683, which results in *P. aeruginosa* strains whose cytotoxicity potential is greatly diminished [31], [37]–[40], [50]. Our structure reveals that the C-terminus of ExoU forms a distinct domain (encompassing residues 588–687) that folds into a 4-helical bundle (red in Figures 1 and 3a). One group of residues whose mutation affects ExoU intracellular functionality (Ile609, Ile654, Leu651, Ala678; [38], [40]) makes hydrophobic interactions within the bundle itself, and their mutation into polar residues could destabilize the non-polar character of the interior of the bundle. A second group (Arg661, Lys679, Arg682, Asn683 [38], [39]) is exposed on the surface of ExoU, with Arg661 located on a loop that precedes the C-terminal α-helix that harbors amino acids 679–683 (Figures 3a and 3b). Interestingly, residues 679, 682, and 683 form a polar, mostly basic ‘backbone’ that could bind to negatively charged eukaryotic phospholipids (Figure S3), and facilitate lipid bilayer recognition. To explore this idea, we performed protein-lipid overlay assays and demonstrated that ExoU strongly interacts with PI(4,5)P_2_ (Figure 3c), a negatively-charged polyvalent phospholipid that is abundant in the cytosolic leaflet of the PM. The non-catalytic mutant ExoU-S142A also displays a strong affinity for PI(4,5)P_2_, but this interaction is completely abolished in the case of a mutant lacking residues 679–683 (Figure 3c), which suggests a direct role of PI(4,5)P_2_ interaction for ExoU PM localization. Our results thus provide a molecular explanation for ExoU's PM targeting, which constitutes the toxin's premier localization upon T3SS translocation.

### Ubiquitinated ExoU is targeted to endosomes

**Figure 4:**
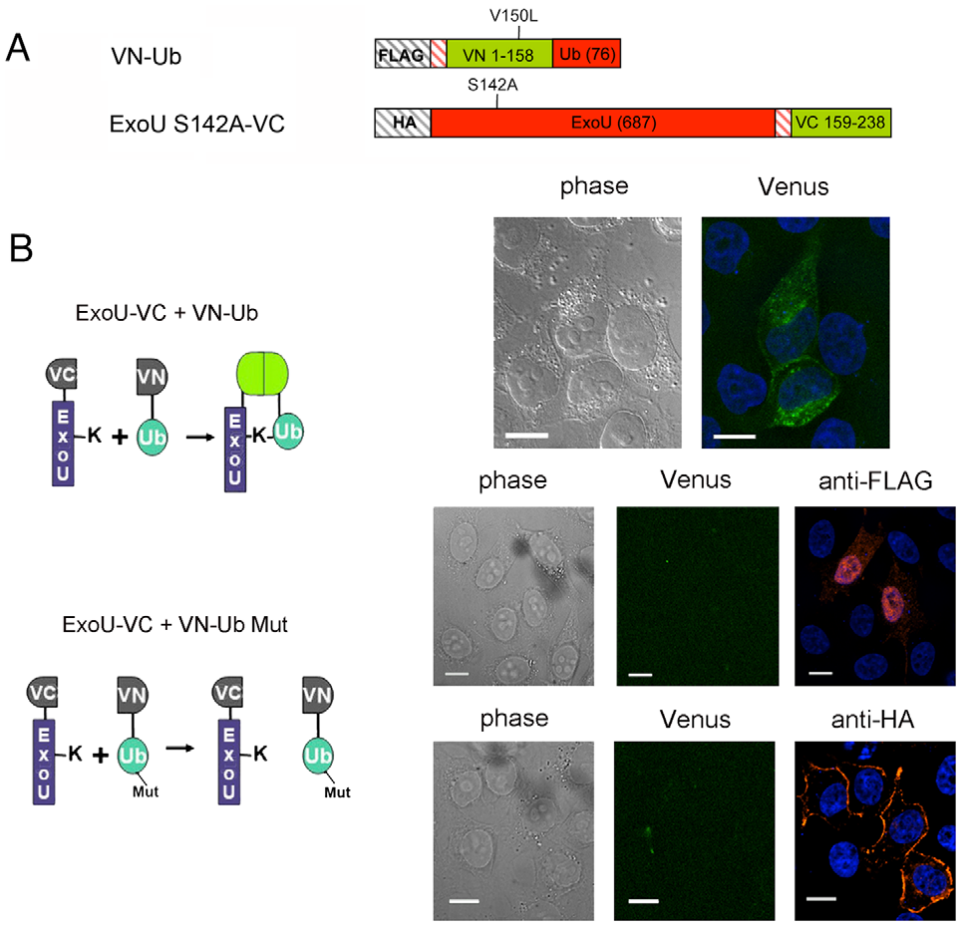
Direct visualization of Ub-ExoU by UbFC. (A) Schematic representation of the plasmid constructs used for UbFC. The genes from which the different fragments were amplified are indicated within the boxes, and the number of residues of each fragment is indicated in parentheses. (B) ExoU-S142A-VC and VN-Ub (upper panel) or ExoU-S142A-VC and VN-Ub Mut (lower panels) were co-expressed in HeLa cells, and fluorescence complementation was observed 36 h after transfection. Immunodetection of fusion proteins with the anti-HA and the anti-FLAG antibodies was performed to confirm protein expression Bars, 12 µm. Pearson's correlation coefficient for Fig. 4B top=0.727.

Upon localization to the PM, ExoU becomes ubiquitinated on Lys178, a modification which has a small effect on its intracellular turnover rate [39]. In order to explore the fate of translocated ExoU, we adapted the Ubiquitin-mediated Fluorescence Complementation (UbFC) method [52] to directly visualize ubiquitinated ExoU (Ub-ExoU) in eukaryotic cells. Catalytically-inactive ExoU-S142A was fused to the C-terminal fragment of the Venus fluorescent protein (generating ExoU-S142A-VC), and Ub to its complementary N-terminal fragment (generating VN-Ub; Figure 4a). Co-transfection of the two plasmids, but not single transfections, led to a fluorescent signal located at the cell periphery, but also revealed punctate structures throughout the cytoplasm (Figure 4b upper panel). To ascertain that the observed signal was specific, we tested the fluorescence complementation potential of a mutated, non-conjugatable Ub protein, in which the 7 endogenous lysine residues and the two C-terminal glycine residues were modified (VN-Ub Mut). No fluorescence complementation was observed when co-transfecting ExoU-S142A-VC and VN-Ub Mut, whereas both proteins were successfully expressed as evidenced by immunofluorescence detection of tagged proteins (Figure 4b, bottom panels). These observations suggested that the punctate pattern observed by Phillips et al upon transfection of GFP-ExoU-S142A into eukaryotic cells [32] could reflect targeting of the ubiquitinated form of ExoU to specific intracellular compartments.

**Figure 5:**
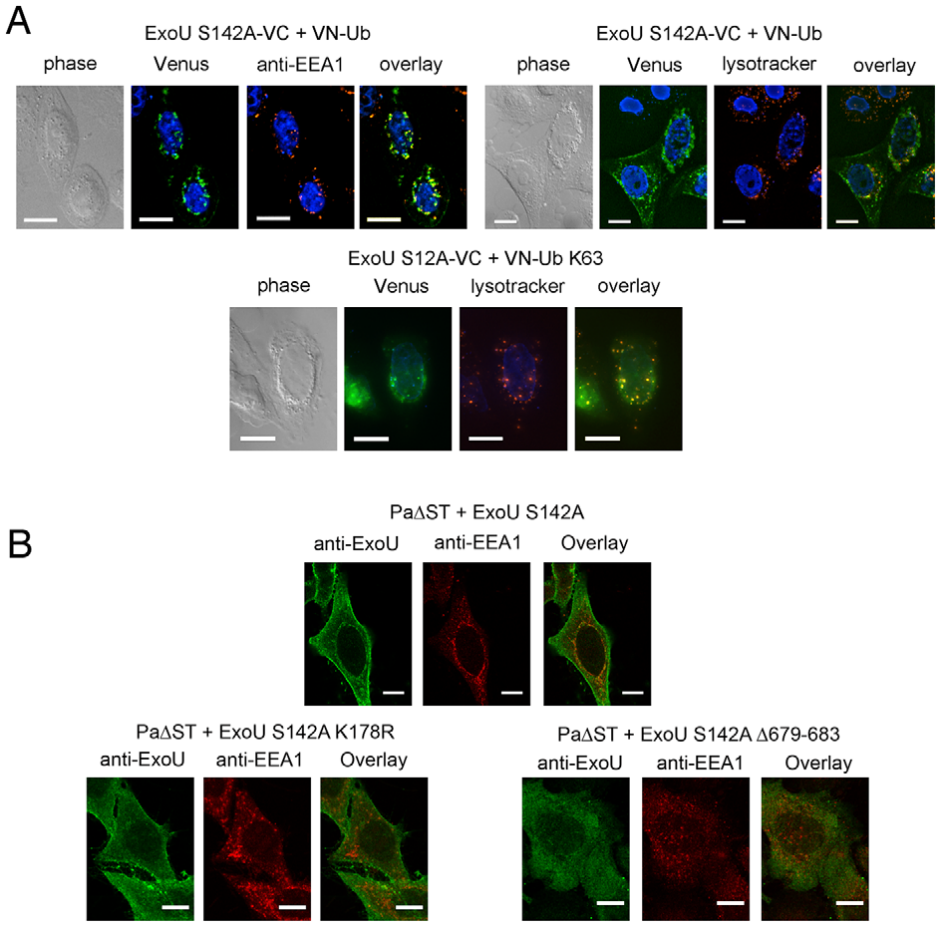
Ub-ExoU is targeted to endosomes. (A) Co-localization of Ub-ExoU with endosomal markers. ExoU S142A-VC was co-transfected with VN-Ub (upper panel) or with VN-Ub K63 (bottom panel). 36 h later, cells were stained with the anti-EEA1, or treated with lysotracker red. Pearson's correlation coefficients correspond to 0.692, 0.570, and 0.801 the top left, top right, and bottom panels, respectively. (B). HeLa cells were infected for 3 h with *Pa*ΔST+ExoU S142A (upper panel), *Pa*ΔST+ExoU S142A-K178R or *Pa*ΔST+ExoU S142A Δ679–683 (bottom panel), fixed and immunolabeled with anti-ExoU and anti-EEA1 antibodies. Single two-channel confocal images are presented. The Pearson's correlation coefficient for EEA1-ExoU=0.213. Bars, 12 µm.

In order to identify this intracellular location, we compared the fluorescence complementation signal with the localization of proteins of known distribution. Ub-ExoU-S142A did not co-localize with endoplasmic reticulum nor with Golgi markers (Figure S4), but was targeted to acidic organelles, as evidenced by its co-localization with the early endosome marker EEA1 and with lysotracker-stained compartments (Figure 5a, top panels). Since ubiquitination of ExoU has been proposed to involve Lys63-linked Ub chains [39], we tested a Venus-fusion of a Ub mutant in which all Lys residues, except Lys63, were mutated to Arg (VN-Ub-K63). ExoU-S142A-Ub K63 co-localized with lysotracker (Figure 5a, bottom panel), confirming that K63-linked chains of Ub are most likely responsible for ExoU targeting to endosomal compartments.

In order to study ExoU targeting in the natural context of infection, we engineered a *P. aeruginosa* strain that translocates catalytically inactive ExoU-S142A upon induction of the T3SS into HeLa cells (PaΔST+ExoU). Confocal microscopy analyses of infected cells revealed the presence of ExoU at the PM, as previously identified [32], [37], [39], but also revealed that translocated ExoU co-localizes with EEA1 (Figure 5b top panel and Video S1). In order to relate this localization to the ubiquitination of ExoU, we generated a *P. aeruginosa* strain that expresses ExoU-S142A-K178R, which cannot be ubiqutinated [39]. Confocal microscopy analyses of HeLa cells infected with this strain did not indicate any co-localization of this mutant with endosomal compartments, but a prominent PM staining. These observations thus indicate that ExoU's co-localization with endosomes is directly dependent on its ubiquitination of Lys178.

Stirling et al. showed that residues 679–683, located at the extremity of the C-terminal helical bundle (Figure 3b) are required for ubiquitination [39]. The intracellular fate of this mutant was investigated by constructing a *P. aeruginosa* strain that expresses ExoU-S142A-Δ679–683. Confocal microscopy images of infected HeLa cells indicated that this mutant form of ExoU not only failed to associate with the PM, but was also not able to co-localize with EEA1 (Figure 5b, bottom right panel). These observations point clearly to the interdependence of the membrane-binding bundle, of Lys178 ubiquitination, and of phospholipase activity in mediating the cellular functionality of ExoU.

## Discussion

The acute cytotoxic potential of ExoU-carrying *P. aeruginosa* strains is evidenced by the significant damage suffered by a variety of cell lineages and the toxin's ability to rapidly destroy lung tissue *in vivo* [24], [25]. The structural and functional characterization of this unique T3SS toxin, reported here, indicates how this multi-domain molecule can intertwine functionalities, and sheds light on its intracellular fate upon translocation.

In the bacterial cytosol and prior to translocation, ExoU is bound to SpcU [23], a small, acidic Type IA T3SS chaperone. Type IA chaperones are typically dimeric, and contact their cognate effectors mostly through the latter's N-terminus, which is ‘wrapped around’ the chaperone dimer [3], [45]–[48], [53]. Here, we identify the two key recognition sites between SpcU and ExoU as the β motif and a hydrophobic cavity at the center of the chaperone, both of which have been described for other Type IA chaperone∶effector complexes, such as InvB∶SipA from *Salmonella* [46], SycE∶YopE (23–78) from *Yersinia* [47], and SicP∶SptP (35–139) from *Salmonella* [48]. In all of these structures, the chaperone-binding domain (CBD) is localized within the effector's N-terminal region. SpcU maintains the N-terminal residues of ExoU in a non-globular conformation (albeit still maintaining the structure of β1). This could allow ExoU to be ready for secretion and rapid unfolding upon recognition by the base of the T3SS, as suggested for other systems [5], [54]. This hypothesis is also supported by mutagenesis studies performed on the β motifs of SicP∶SptP and SycE∶YopE, which reveal that modification of a few strategic residues suffices for total disruption of chaperone∶effector interactions [46].

The structure of the phospholipase domain of ExoU reveals that the catalytic Ser142 is not exposed on the surface of the molecule, but is located within a cleft surrounded by flexible regions. This suggests that the active site does not have unobstructed access to its phospholipidic substrates. Local conformational changes could thus be required to occur upon membrane targeting, leading to active site exposure, which could constitute a potential mechanism of protection of membranes (bacterial or host) that are not originally intended for targeting. Notably, the localization of a phospholipase active site serine residue within a funnel-like region protected by a flexible lid carrying the catalytic aspartate has been observed in the structure of the eukaryotic enzyme cPLA_2_ [51], which undergoes interfacial activation upon binding to a membranous interface [55], [56]. In cPLA_2_, closure of the lid region is necessary not only for formation of a ‘complete’ active site, but also for masking of a large hydrophobic region that surrounds the catalytic serine [51]. In ExoU, the localization of Asp344, the second member of the catalytic dyad, in a flexible region in close proximity to Ser142, provides additional supportive evidence for the requirement for local conformational changes in the vicinity of Ser142 in order to place Asp344 in position for catalysis. This suggests that the structure presented here is in an ‘open’, inactive conformation, and local conformational changes are required to ‘close’ the flexible loops and define the placement of Asp344.

How could such conformational modifications be initiated? The data presented here, including our structural analysis and phospholipid binding studies, together with mutagenesis results, spectroscopic studies, and cofactor identification experiments performed by other laboratories [31]–[33], [37], [38], [40]–[44] provide a working hypothesis. ExoU binds specifically to PI(4,5)P_2_, the major phosphoinositide of the eukaryotic plasma membrane, and the C-terminal α-helical bundle plays a key role in this association (Figure 3c). Notably, this C-terminal region is of central importance for ExoU's phospholipase activity [31], [38]–[40], an observation that reveals the functional interdependence of membrane-binding and catalytic domains. Thus, upon translocation of ExoU into target cells and PM recognition by the C-terminal domain, ExoU could undergo a conformational change that allows partial insertion of the nearby catalytic domain, and thus the active site, into the bilayer. The proximity of the substrate, and the interaction with a eukaryotic cofactor, could suffice to locally modify the flexible regions that surround the active site region, allowing the catalytic serine to have access to phospholipids. The distance between the C-terminus of ExoU and the active site is of approximately 25 Å, and the lack of many contact points between these two domains suggests that some level of rotation could occur between them in order to place the active site in an optimal position. This suggestion is also in agreement with recent, elegant studies by Benson et al., who used spin-labeling electron paramagnetic resonance spectroscopy to identify conformational changes in the C-terminus domain and within the active site of ExoU, in the presence of both liposomes and of a potential eukaryotic cofactor [43]. Strikingly, Benson et al. also identified that in the absence of cofactor or lipids, ExoU displays multiple conformations, which is in particular agreement with the considerable flexibility observed in the region of the active site in our apo structure. In another recent study, ubiquitin and ubiquitinated proteins were also identified as cofactors, and potential activating agents, for ExoU [44]. Considering that many ubiquitination processes occur in the vicinity of the PM, these observations are also in agreement with the model discussed above.

ExoU has been reported to be ubiquitinated by the host machinery on its Lys178 upon translocation and binding to the PM [39]. Our structure reveals that Lys178 is located within the catalytic domain, on the same side of the molecule as both the active site and the membrane-binding domain (Figures 3a and 3b). Following the model described above, the partial imbedding of the catalytic domain of ExoU into the bilayer (upon recognition of the PM through the C-terminal region and binding to cofactor(s)) would place Lys178 in close proximity to ubiquitin ligase complexes located in the vicinity [57], allowing for rapid ubiquitination. Notably, we show that ubiquitinated ExoU is observed at the level of the organelles of the endo-lysosomal pathway, both by transfection studies using fluorescence complementation, and confocal microscopy analyses of *P. aeruginosa*-infected eukaryotic cells. It has been previously shown that a *P. aeruginosa* strain carrying the non-ubiquitinated variant ExoU-K178R is as cytotoxic to eukaryotic cells as the wild type strain [39], suggesting that endosomal localization is not linked to ExoU's cytotoxic potential. Rather, the effects described here could represent an attempt by the eukaryotic cell to protect itself from ExoU's phospholipase action by targeting it to lysosomes, viewing its potential destruction. Given the rapid effect of ExoU on target cells, however, this protection mechanism does not seem to be efficient enough to offer protection from the toxin's ability to efficiently disrupt membranes, which is at the basis for the extremely aggressive nature of ExoU-expressing *P. aeruginosa* strains.

In summary, the structure of ExoU presented here reveals the toxin in its chaperone-bound, inactive, pre-secretion conformation. Since ExoU has broad substrate specificity, this conformation most likely prevents the toxin from binding to bacterial membranes, enabling the bacterium to be protected from ExoU's phospholipase action. Thus, our structural data provide a basis for the study of conformational changes that must accompany ExoU's activation upon binding to cellular factors, which in turn lead to phospholipase activity and cytotoxicity. Finally, the structure of the ExoU∶SpcU complex could serve as a basis for the development of ligands designed to stabilize this conformation, which in turn could prevent ExoU's secretion and thus diminish bacterial pathogenicity.

## Methods and Materials

### Protein expression, crystallization, and structure solution

ExoU (1–687) and SpcU (1–137) were first amplified from a clinical *P. aeruginosa* strain (GESPA 1999 collection). Both full-length forms of ExoU and SpcU were initially cloned into pETDueT-1, co-expressed in *E. coli* BL21/DE3, and expression was performed with 1 mM isopropyl 1-thio-β-D-thiogalactopyranoside for 3 h at 37°C. The complex was purified by nickel affinity chromatography (Ni-NTA, Qiagen) and gel filtration (HR 10/60 column, GE Healthcare) techniques. This strategy yielded thin crystals that diffracted poorly. Limited proteolysis experiments with thrombin identified that removal of the first 29 residues of ExoU and the last 10 residues of SpcU generated better quality crystals. Thus a thrombin site was introduced between residues 29 and 30 of ExoU, and a stop codon after residue 127 of SpcU, in the same pETDueT vector. The complex was expressed as described above. After Ni affinity chromatography and treatment with thrombin, ExoU∶SpcU was further purified a MonoQ column (GE Healthcare) in 50 mM Tris-HCl pH 8.8, 1 mM EDTA (elution gradient, 0–1 M NaCl). Samples for crystallization were gel filtered in 25 mM Tris-HCl pH 8.0, 100 mM NaCl, 1 mM EDTA. Crystals of the ExoU (29–687)∶SpcU (1–127) complex were obtained in 0.1 M KNO_3_, 20% PEG 3350. A SAD data set on the selenium edge was collected at the European Synchrotron Radiation Facility (ESRF) ID29 beamline (Grenoble, France) to a resolution of 2.94 Å. Data collection and refinement statistics are included in Table S1.

Diffraction images were indexed and scaled with XDS [58]. Initial selenomethionine sites were identified with PHENIX AutoSol [59]. Initial experimental density maps were improved after model building and phase extension using PHENIX. PARROT [60] and PIRATE [61]were used in density modification-phase improvement steps interspersed with cycles of automatic model-building using BUCCANEER [62]. The ExoU∶SpcU structure was completed by cycles of manual model-building using COOT 0.6.2 [63]. Water molecules were added to the residual electron density map using ARP/wARP 7.1.1 [64]. Cycles of restrained refinement were performed with REFMAC 5.6 [65] as implemented in the CCP4 program suite (Table S1). Stereochemical verification was performed by PROCHECK [66] and secondary structure assignment by DSSP [67]. Figures were generated with PyMol (www.pymol.org).

### Mass spectrometry

Native mass spectrometry measurements of ExoU∶SpcU were carried out with a Micro-TOF-Q Bruker mass spectrometer (Wissembourg, France) with an electrospray ion source. Mass spectra were recorded in the 500–7000 mass-to-charge (*m/z*) range. Sample concentration was 15 µm in 20 mm ammonium acetate and continuously infused at a flow rate of 7 µl/min. Data were acquired in the positive mode and calibration was performed using a solution of 0.1 mg/ml CsI in water/isopropyl alcohol (1∶1, v/v). The system was controlled with the MicrOTOF Control software package and data were processed with DataAnalysis.

### Construction of plasmids for transfection of HeLa cells

Plasmids encoding ExoU S142A-VC and VN-Ub originate from Addgene plasmids 22011 and 22010, initially provided by Dr. C.-D. Hu, Purdue Univ. ExoU was cloned N-terminally to the VC155 fragment, whereas Ub was cloned C-terminally to the VN173 fragment. The S142A mutation was inserted into the ExoU sequence via site-directed mutagenesis (Stratagene). To limit background fluorescence [68], we deleted specific regions of the Venus fragments to generate VN158-Ub and ExoU-S142A-VC159, and introduced the V150L mutation in the VN fragment, to obtain plasmids ExoU-S142A-VC and VN-Ub as described in [69]. We amplified Ub K63 from Addgene plasmid 17606 (donated by Dr T. Dawson), and cloned it in our modified pBiFC-VN plasmid. Insertion of the Lys63Arg, Gly75Lys and Gly76Leu substitutions led to the control plasmid VN-Ub Mut.

### Construction of mutant ExoU-expressing *P. aeruginosa* strains

A 3-kb-long DNA fragment containing the promoter and the sequence of the *exoUspcU* operon from the strain of the GESPA collection [70] was cloned into the replicative pUCP20 plasmid. The S124A and K178R mutations, as well as the 679–683 deletion were introduced into the *exou* sequence using Quick Change Mutagenesis kit (Stratagene). The different plasmids were transferred by transformation [71] into the *P. aeruginosa* CHAΔST*lox* strain kindly provided by Prof B. Polack.

### Antibodies and probes

Antibodies used for immunofluorescence co-staining include an anti-HA from Covance (AFC-101P), an anti-FLAG from Euromedex (EL1B11), an anti-EEA1 from BD transduction (610457) and an anti-GORASP2 from Euromedex (10598-1-AP). The anti-ExoU antibodies were raised against His_6_-ExoU in rabbits by Eurogentec, as described by the manufacturer. Specific anti-ExoU antibodies were immunopurified. Lysotracker Red (Molecular probes) was added at 500 nM and incubated for 1 h at 37°C before fixation.

### Mammalian cell transfections and immunofluorescence microscopy

One day before transfection, Hela cells were seeded on 12-mm coverslips. Transient transfections were performed using Jetprime (Polyplus transfection), following the manufacturer's instructions. For bifluorescence complementation assays, both plasmids were added in equal quantities. On the following day, cells were fixed with 2% paraformaldehyde and permeabilized with 0.2% TX-100. Primary antibodies were added for 30 min at 37°C, washed away and the Alexa-fluor 594 conjugated anti-mouse antibody (Molecular Probes) was incubated for 1 h at room temperature. Nuclei were counterstained with DAPI. Images were collected with an inverted Olympus IX81 epifluorescence motorized microscope equipped with a motorized piezo stage (Ludl Electronic Products, USA) and a Retiga-SRV CCD camera (QImaging) driven by VOLOCITY software (Improvision) with a binning of 1, using a PlanApo 60× NA1.42 objective (Olympus). Images were deconvoluted using the Volocity 5.5 software.

### *P. aeruginosa* infection and analysis by confocal microscopy

Sparse HeLa cells were infected with bacteria in exponential growth at an MOI value of 10 and incubated for 3 h at 37°C in RPMI. At this point, approximately 10% of the total amount of ExoU is ubiquitinated. Subsequently, cells were washed and fixed in 4% paraformaldehyde and 0.5% Triton X-100 and labeled with anti-ExoU and anti-EEA1 antibodies. After incubation with secondary fluorescent antibodies, cells were observed with a confocal fluorescent microscope (Leica TCS SP2). Images were treated with the ImageJ software for 3D reconstruction.

### Detection of protein-lipid interactions

Membrane lipid strips (Echelon Biosciences) were blocked in PBS+4% milk, and incubated overnight with proteins at 0.5 µg/mL. Detection of bound proteins was performed as recommended by the manufacturer.

## Supporting Information

Figure S1 **Association between ExoU and its chaperone SpcU.** (A) SpcU has a type IA T3SS chaperone fold whose central β-sheet is completed by one β-strand from the effector molecule, ExoU, forming a 6-stranded structure. The SpcU ‘tunnel’ that harbors the ExoU's β1 strand is highly hydrophobic, as seen in the electrostatic surface representation in (B), where basic residues are shown in blue and acidic in red. An additional, minor interaction region involves the membrane binding domain, in which the backbone carboxyl group of SpcU's Val49 and the side chain carbonyl group of Ser51 make hydrogen bonds with the NH2 group of Arg633 and the ND2 moiety of Asn657, respectively. (C) A symmetry mate within the C2 cell reveals the formation of a SpcU dimer that is wrapped by the N-terminus of ExoU.
(TIF)

Figure S2 **The bridging domain of ExoU is subdivided into two subdomains.** The N-terminal subdomain harbors a 4-stranded region formed by strands that correspond to sequences both N- and C-terminal to the catalytic domain. The second subdomain, fully helical, is composed of residues 481–580.
(TIF)

Figure S3 **Electrostatic surface diagram of ExoU, with a direct view to the C-terminus domain.** Residues 679–683 are not only completely solvent exposed but also generate a polar/basic ‘backbone’ that could recognize the phospholipid bilayer.
(TIF)

Figure S4 **Ub-ExoU is not targeted to the Golgi.** 36 h after co-transfection of ExoU-VC and VN-Ub, cells were stained with an anti-GORASP2 antibody to label the cis-Golgi. The Person's correlation coefficient corresponds to 0.0546. Bars, 12 µm.
(TIF)

Table S1 **Data collection and structure refinement statistics.**
(TIF)

Video S1 **Co-localization of ExoU and EEA1 in a stack of Z-sections of infected HeLa cells.** Images were acquired on a confocal microscope for ExoU (red) and EEA1 (green) immunofluorescence and collected in 22 Z-sections. Co-localization in early endosomes appears in yellow.
(AVI)
