# Supplementary figures and images for: Structural Basis of Cytotoxicity Mediated by the Type III Secretion Toxin ExoU from Pseudomonas aeruginosa
Source: PLoS Pathog. 2012 Apr 5;8(4):e1002637. doi: 10.1371/journal.ppat.1002637 (PMC3320612; doi:10.1371/journal.ppat.1002637)

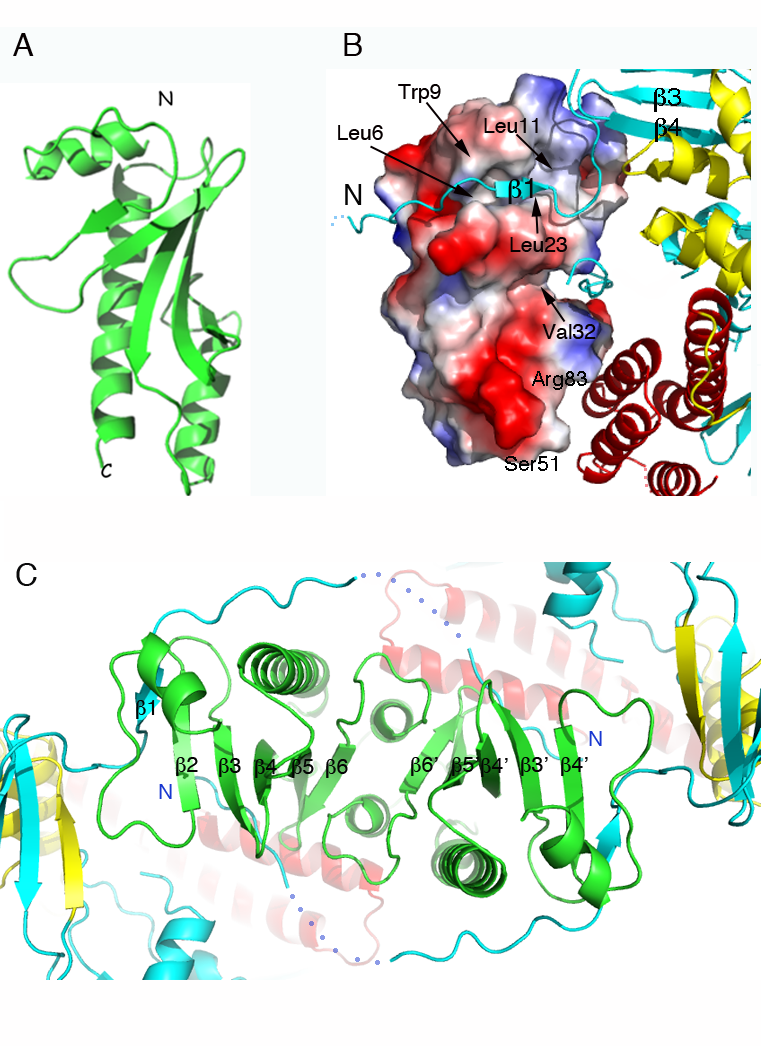

Supplement: Figure S1 — Association between ExoU and its chaperone SpcU. (A) SpcU has a type IA T3SS chaperone fold whose central β-sheet is completed by one β-strand from the effector molecule, ExoU, forming a 6-stranded structure. The SpcU ‘tunnel’ that harbors the ExoU's β1 strand is highly hydrophobic, as seen in the electrostatic surface representation in (B), where basic residues are shown in blue and acidic in red. An additional, minor interaction region involves the membrane binding domain, in which the backbone carboxyl group of SpcU's Val49 and the side chain carbonyl group of Ser51 make hydrogen bonds with the NH2 group of Arg633 and the ND2 moiety of Asn657, respectively. (C) A symmetry mate within the C2 cell reveals the formation of a SpcU dimer that is wrapped by the N-terminus of ExoU. (TIF) [file ppat.1002637.s001.tif]

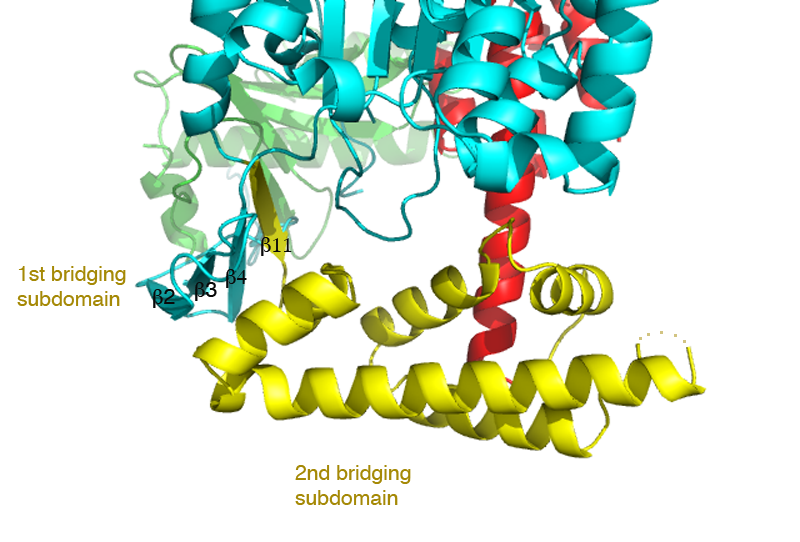

Supplement: Figure S2 — The bridging domain of ExoU is subdivided into two subdomains. The N-terminal subdomain harbors a 4-stranded region formed by strands that correspond to sequences both N- and C-terminal to the catalytic domain. The second subdomain, fully helical, is composed of residues 481–580. (TIF) [file ppat.1002637.s002.tif]

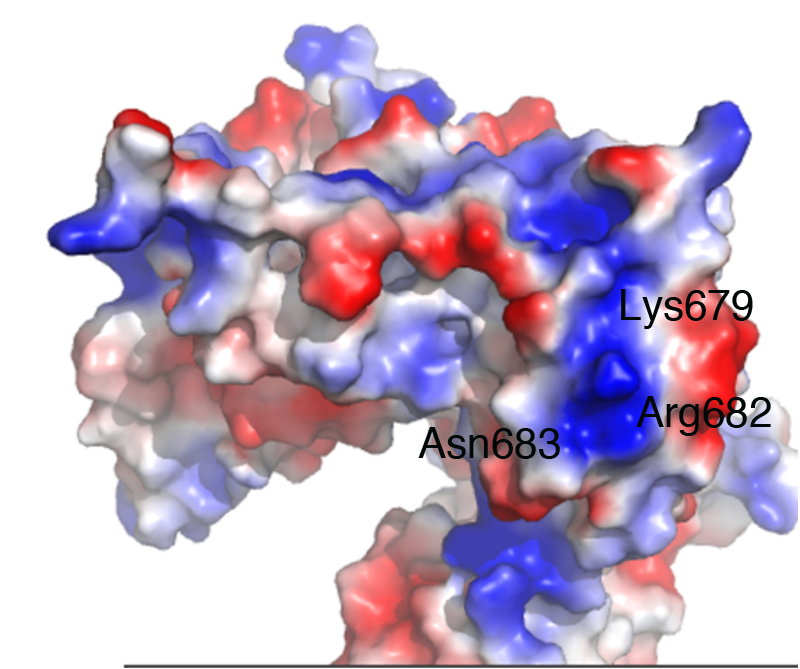

Supplement: Figure S3 — Electrostatic surface diagram of ExoU, with a direct view to the C-terminus domain. Residues 679–683 are not only completely solvent exposed but also generate a polar/basic ‘backbone’ that could recognize the phospholipid bilayer. (TIF) [file ppat.1002637.s003.tif]

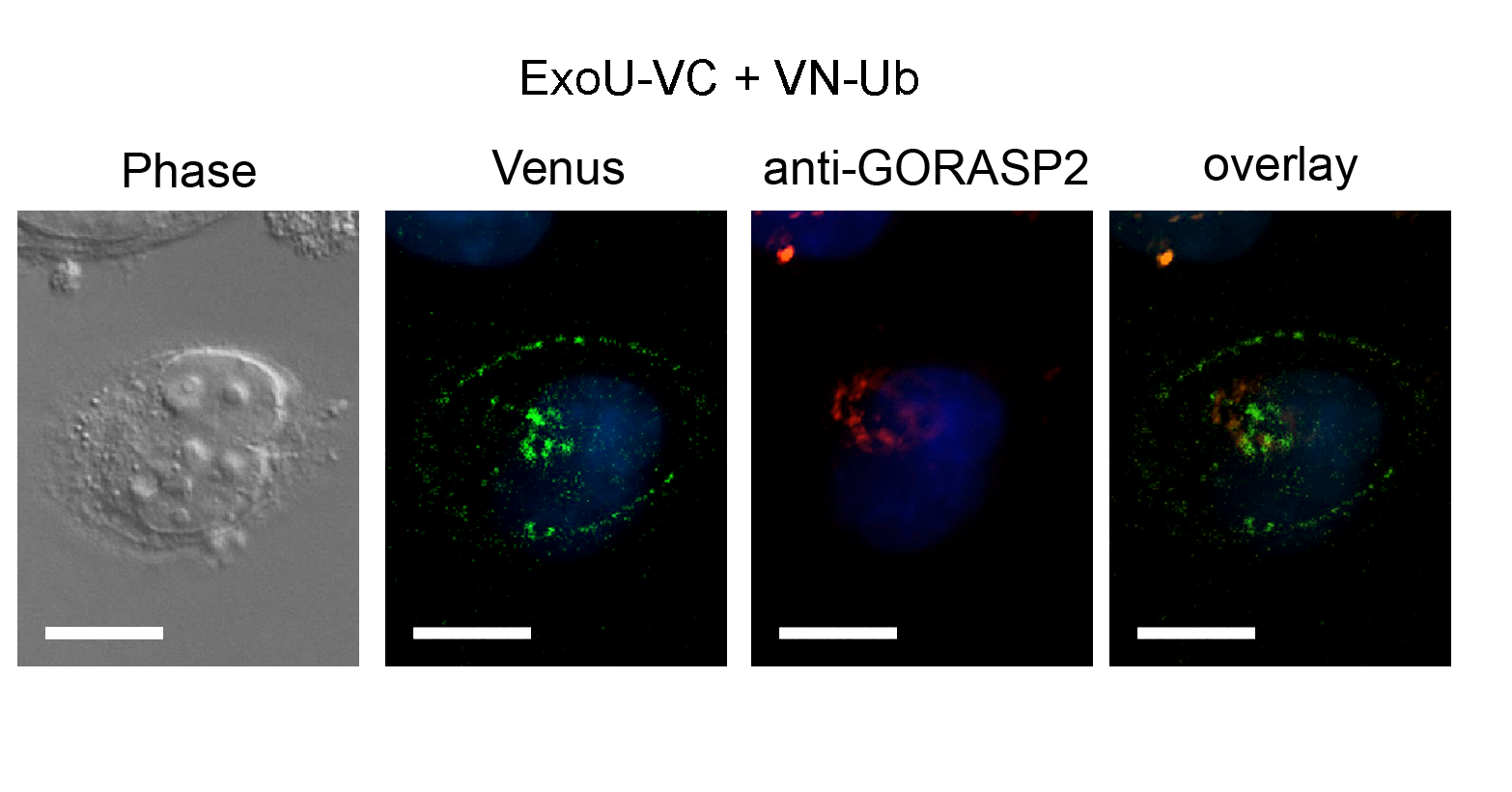

Supplement: Figure S4 — Ub-ExoU is not targeted to the Golgi. 36 h after co-transfection of ExoU-VC and VN-Ub, cells were stained with an anti-GORASP2 antibody to label the cis-Golgi. The Person's correlation coefficient corresponds to 0.0546. Bars, 12 µm. (TIF) [file ppat.1002637.s004.tif]

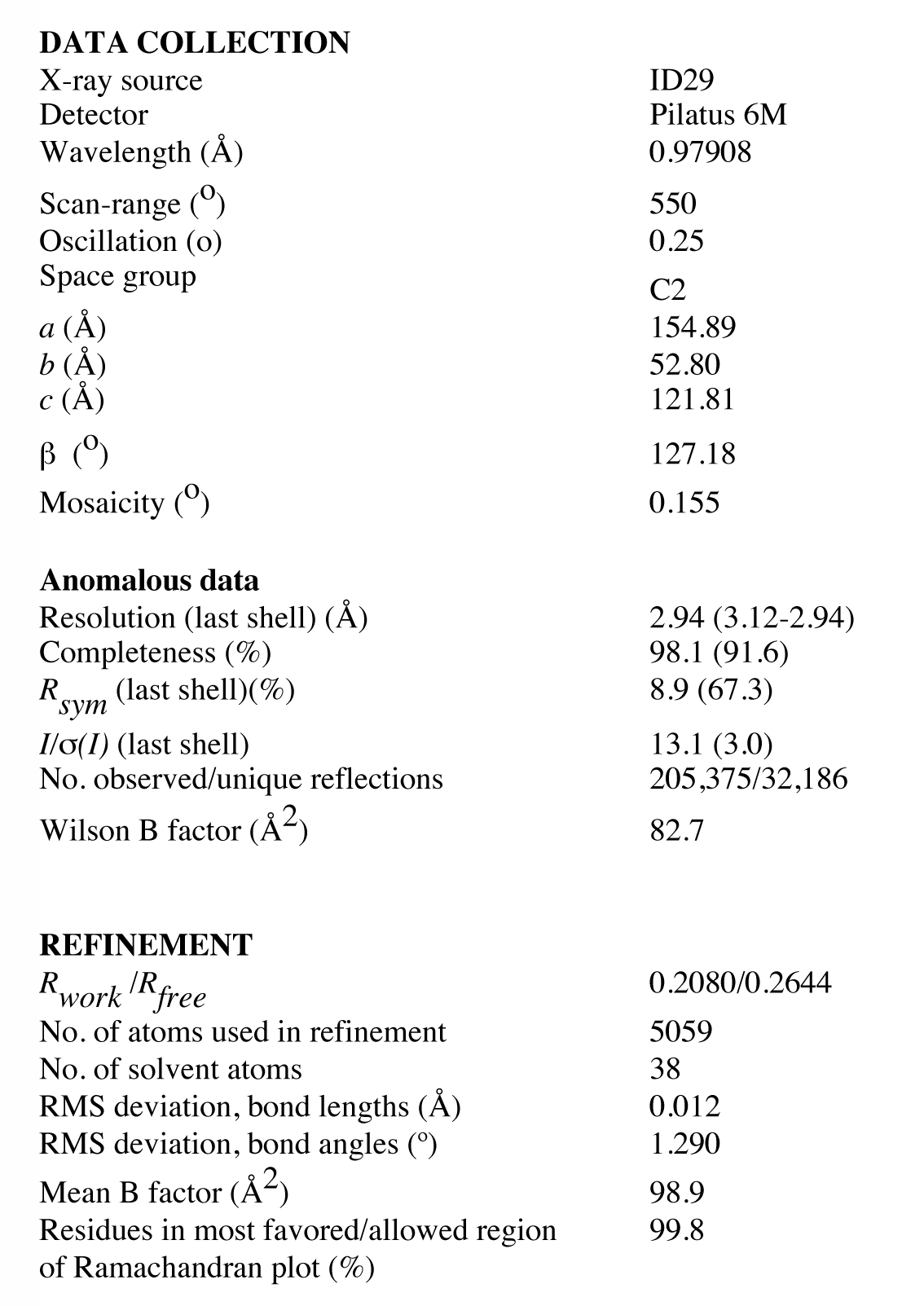

Supplement: Table S1 — Data collection and structure refinement statistics. (TIF) [file ppat.1002637.s005.tif]
